# Exploring Eye Movements in Patients with Glaucoma When Viewing a Driving Scene

**DOI:** 10.1371/journal.pone.0009710

**Published:** 2010-03-16

**Authors:** David P. Crabb, Nicholas D. Smith, Franziska G. Rauscher, Catharine M. Chisholm, John L. Barbur, David F. Edgar, David F. Garway-Heath

**Affiliations:** 1 Department of Optometry and Visual Science, City University London, London, United Kingdom; 2 Bradford School of Optometry and Vision Science, University of Bradford, Bradford, United Kingdom; 3 NIHR Biomedical Research Centre for Ophthalmology, Moorfields Eye Hospital NHS Foundation Trust and UCL Institute of Ophthalmology, London, United Kingdom; University of Minnesota, United States of America

## Abstract

**Background:**

Glaucoma is a progressive eye disease and a leading cause of visual disability. Automated assessment of the visual field determines the different stages in the disease process: it would be desirable to link these measurements taken in the clinic with patient's actual function, or establish if patients compensate for their restricted field of view when performing everyday tasks. Hence, this study investigated eye movements in glaucomatous patients when viewing driving scenes in a hazard perception test (HPT).

**Methodology/Principal Findings:**

The HPT is a component of the UK driving licence test consisting of a series of short film clips of various traffic scenes viewed from the driver's perspective each containing hazardous situations that require the camera car to change direction or slow down. Data from nine glaucomatous patients with binocular visual field defects and ten age-matched control subjects were considered (all experienced drivers). Each subject viewed 26 different films with eye movements simultaneously monitored by an eye tracker. Computer software was purpose written to pre-process the data, co-register it to the film clips and to quantify eye movements and point-of-regard (using a dynamic bivariate contour ellipse analysis). On average, and across all HPT films, patients exhibited different eye movement characteristics to controls making, for example, significantly more saccades (P<0.001; 95% confidence interval for mean increase: 9.2 to 22.4%). Whilst the average region of ‘point-of-regard’ of the patients did not differ significantly from the controls, there were revealing cases where patients failed to see a hazard in relation to their binocular visual field defect.

**Conclusions/Significance:**

Characteristics of eye movement patterns in patients with bilateral glaucoma can differ significantly from age-matched controls when viewing a traffic scene. Further studies of eye movements made by glaucomatous patients could provide useful information about the definition of the visual field component required for fitness to drive.

## Introduction

Glaucoma is a chronic progressive neuropathy involving the visual pathway: it is the most common cause of irreversible blindness in the world and a leading cause of visual morbidity. Given its chronic progressive nature, the rise in prevalence with age and the increasing longevity of the population, the impact of the disease is substantial and rising.

Automated visual field assessment is the principal functional measurement in the clinical management of patients with glaucoma. However, insight into how visual field defects manifesting at different stages in the disease process impact on patients' everyday visual function is only recently gaining momentum in the research literature. Linking the clinical measurements to what patients can do functionally is important in the management of the condition. For example, there is emerging evidence of glaucomatous patients being at greater risk of falls and accidents, even with relatively modest visual field defects [1], [2], [3]. There is also evidence of the impact of glaucomatous field defects on self-reported disability [4], [5], [6], [7], [8] and, more recently, objective measures in laboratory based studies demonstrate the difficulty patients have with some everyday tasks [9], [10]. Glaucoma undoubtedly impacts on an individual when visual field loss causes the removal of a driving licence, and several studies of varying experimental design have shown that certain glaucomatous visual field defects are not compatible with safe driving [1], [11], [12], [13]. The issue of driving with glaucoma is a serious one, with a significant proportion of patients suffering visual loss that results in driving ineligibility, certainly in the UK [14], [15]. Moreover, it has been demonstrated that patients perceive this particular potential outcome of their disease to be as serious to them as the long-term risk of blindness [16].

One aspect of visual function in glaucoma that has received little attention is eye movements. It is accepted that patients' perception of the severity of their peripheral visual field loss is influenced by one eye essentially ‘filling in’ for the other, but very little is known about how eye movements might contribute to any compensatory mechanism for loss of function. It would, for example, be useful to know if glaucoma patients exhibit the same eye movement behaviour as healthy subjects, or maintain the same point of regard when looking at, or interpreting, an everyday scene. This evidence may inform future studies that attempt to relate the severity, extent and location of a visual field defect to what a patient can and cannot do in terms of real life activities, especially driving. It may even provide a basis for incorporating attention to eye movements into visual rehabilitation and coping strategies for the patient with glaucoma.

The Hazard Perception Test (HPT) was introduced as an element of the UK driving licence theory test in 2003 as a measure to encourage appropriate visual scanning of the road or highway and to develop the ability to recognise at the earliest opportunity that a potentially dangerous driving situation might arise. In this context a ‘learner driver’ is shown a film of a real driving scene, seen from the perspective of the driver, with the task being to detect potential ‘hazards’: these are defined as something that would make the camera car take evasive action, such as braking for an oncoming cyclist or a pedestrian unexpectedly crossing the road/street. Primarily, it is a useful educational tool allowing learner drivers to encounter ‘on road’ driving scenes from the safety of a computer monitor. A subject's performance on the HPT is clearly dependent on instruction, prior experience and perception of what represents a driving hazard, and HPT performance in terms of simply detecting a hazard in visually healthy subjects is certainly variable [17]. Previously the HPT, as a simple reaction test to hazards, was investigated as a potential proxy measure for identifying visual defects that would impinge on fitness to drive, and was found to have limited value for this purpose [18]. Whether the HPT is an indicator of subjects' visual strategies when driving under the conditions in which they are most likely to have an accident is open to debate, and is not the subject of this study. Rather, for the purposes of this study, this realistic and relevant visual scenario can be ‘played out’ on a computer screen allowing for accurate gaze tracking in a controlled laboratory setting, thus making it most suitable for examining the eye movement behaviour of glaucomatous patients compared to control subjects whilst viewing an everyday scene.

The aim of this study is to perform eye tracking on subjects as they simultaneously view a series of HPT film clips to examine the hypothesis that patients with binocular glaucomatous field defects exhibit significant differences in eye movement characteristics to healthy control subjects. Moreover, in the context of visual field defects and fitness to drive, the data can also be used to examine qualitatively how these patients' eye movements relate to the events and hazards in the video sequences.

## Materials and Methods

### Ethics Statement

The study was approved by the ethics committee of each of the participating institutions where subjects were recruited (Moorfields and Whittington Research Ethics Committee, London; School of Community and Health Sciences Research and Ethics Committee, City University London). All participants were asked about their general health and were excluded if they were on any significant medication (other than that for their glaucoma). Written informed consent, according to the tenets of the Declaration of Helsinki, was obtained prior to examination from each subject. All the data, with patient identifiers removed, were transferred to a secure computer at the university.

### Subjects

This study took advantage of some of the data collected as part of a UK Department for Transport (DfT) funded study conducted at City University London, fully reported elsewhere [17], [18]. In short, this UK DfT study aimed to examine the agreement between the outcome of conventional visual field tests and proxy measures of driving safety in subjects with binocular paracentral scotoma, resulting from a range of visual disorders, but preserved visual acuity (VA). One experiment in the original study, not performed on all study participants, examined the performance of subjects on the HPT whilst eye movements were simultaneously recorded. Glaucoma patients were recruited from the Central Middlesex Hospital (North West London Hospitals NHS Trust), Moorfields Eye Hospital Trust London and the Fight for Sight Optometry Clinic at City University London. Data from these patients, along with those from a group of visually healthy control subjects, are the subject of this study.

All patients had a clinical diagnosis of glaucomatous optic neuropathy (primary open angle glaucoma) in both eyes and are, therefore, representative of those patients who should undergo further testing to satisfy the visual field requirements for legal fitness to drive in the UK [17], [19]. Prior to inclusion in the study, all patients had a full eye examination including central visual fields (24-2 or 30-2 SITA Standard) recorded on a Humphrey Visual Field Analyzer (HFA, Carl Zeiss Meditec, CA, USA). Patients were excluded if they produced unreliable fields at this first visit. VA of at least 6/9 was required in both eyes, with no other significant ocular disease reported other than glaucoma. Visually healthy control subjects were recruited from University staff, centres for the elderly and the University optometry clinic. A full eye examination, including HFA visual fields, was carried out to exclude any abnormality before a volunteer was accepted into the study. A VA of at least 6/9 was required for each eye.

The HPT film clips used in this study have been used elsewhere [20] and consisted of 26 short films (range 40–73 seconds) showing a range of driving environments (e.g. dual-carriageway or divided highway, rural lane, busy urban streets). Each film contained between one and three ‘hazardous driving events’. The films were digitised (with a standard mpeg codec running at 25 frames per second) and shown on a 42 inch plasma widescreen monitor (1280 by 1024 pixel resolution) in a rectangle of width 824 mm and height 512 mm. Participants were seated at a chin rest 120 cm from the screen and the image subtended 39° horizontally and 25° vertically. Eye movements were monitored using a SMI Eyelink eye tracker (SensoMotoric Instruments, Teltow, Berlin, Germany) sampling at 250 Hz. The SMI eye tracker has reported spatial accuracy better than 0.5°. Calibration, drift correction, and validation were performed using the algorithms provided by the instrument. In this system, adjustment of the measurements for head position is carried out automatically using detectors in the four corners of the monitor, but a chin rest was also used to stabilise head movements and ensure a constant viewing distance.

Participants were all given the same verbal instructions: that they should look out for events that would cause the camera-car to take evasive action such as braking or evasive steering. They held a response button in their preferred hand, and were instructed to press the button whenever they judged one of these hazards as imminent. This ‘press button’ performance in the actual hazard detection task was not considered in this study because previous analysis indicated large variability in ‘press button’ scores and the influence of subject specific definition of what constitutes a hazard [18]. Following calibration of the eye tracker, each subject was shown two introductory films prior to the commencement of data collection, to allow them to familiarise themselves with the task. The film clips were presented in random order with occasional rest periods between films.

Participants were asked to estimate the number of years they had been actively driving. For this study, participants were excluded if they had never held a valid driving licence or had less than 5 years driving experience. Moreover, for this study analysis was restricted to participants that were 50 years of older at the time of the study to ensure that the mean and distribution of age was matched between participants with glaucoma and controls. None of the participants had previously performed the HPT and were therefore naïve to the task.

### Analysis

The eye tracker yields an enormous amount of raw data: essentially a trace of ‘gaze’ recorded as (x,y) coordinates related to the viewing area every 4.2ms for each film. The results from this experiment contained more than six million of these data points. A bespoke application written in Microsoft Visual C# was developed for analysing these time series [21] and this is freely available from the authors; what follows is a short description of the techniques employed in the main analysis.

Eye movements were divided into fixations, saccades and ‘smooth pursuits’ using a velocity based criterion. One difficulty is correctly separating these movements from the variability in the measurement process. Therefore, a noise removal technique was first applied to the data which filters out highly variable non-physiological measurements (high frequency noise) by taking a window or block of 50 (x,y) coordinate eye gaze positions (around 210ms) and calculates the speed of any eye movement between the positions recorded at time point t and t+1. If more than 75% of the recordings within that window move at 30°/s or more then the whole window is removed from the trace because these measurements are assumed less likely to be physiological movements and are more likely attributed to measurement variability. The window moves along the trace to perform the same procedure on the next 50 samples. On completion, an eight sample median filter is passed over the remaining data to remove or dampen ‘low frequency’ noise. Blinks (denoted by zero value data), gaze outside the viewing area and other eye tracking failures were identified then removed from the traces. Films in which less than 40% of the recordings remained were discarded to ensure that we were not removing too much data per film, following a previously used protocol [22].

Once the data are filtered, saccades were defined as the proportion of the trace where the velocity of the eye movement is faster than 30°/s. This criterion of 30°/s or higher for identifying saccades is common in eye movement studies and eye tracking instrumentation, since this is considered the upper limit of pursuit eye movement speed.[23], [24], [25] Any saccade exceeding a duration of 120 ms or is less than 10 ms were excluded from the analysis. In addition saccades that originate or terminate outside the confines of the dimensions of the display of the film (calibration errors) were excluded from the analysis. Fixations were defined as the proportion of the trace where gaze was ‘still’ and the velocity of the eye movement is less than 1.5°/s. We defined periods of the trace where the speed was greater that 1.5°/s but less that 30°/s to be ‘pursuit’ movements, where gaze was most likely ‘tracking’ a moving object in the film. In truth, these pursuit movements are very difficult to quantify even with a sophisticated eye tracker operating at a high frame rate, but we shall refer to them as possible ‘smooth pursuits’ lacking the stability characteristic of a fixation, but without being fast enough to be classified as a physiological saccade. The algorithm then automatically records the number of fixations, the number of saccades and the number of smooth pursuits per second of film. It also yields average fixation duration (ms), average ‘smooth pursuit’ duration (ms) and average saccade amplitude (size in degrees). These six summary eye movement parameters were recorded for each film as viewed by each subject.

The experimental design aimed to minimize ‘the between subject measurement variability’ in the eye movement parameters with all participants viewing the same films, so these were assumed to be the blocks in the experiment. A General Linear Model (GLM) was used to perform univariate analysis of variance (ANOVA) with an unbalanced design (accommodating missing data by the method of median imputation) to assess each of six different eye movement parameters (response variables) using the statistical software package Minitab v.14 (Minitab Inc., Pennsylvania, USA). In this two way ANOVA arrangement, variation in the response variable (each eye movement parameter) was expected to be different across the films and across the subjects, with the null hypothesis of real interest being no difference in the average value for the eye movement parameters between the patients and controls examined (F test on the main factor, participant group, from the ANOVA).

In order to consider the hypothesis that any differences in eye movement characteristics between patients and controls were similar at the time of the hazards compared to sequences of the HPT where no hazards occurred, the films were divided into ‘safe’ and ‘unsafe’ segments. One of the authors (NS) carefully reviewed all the films and identified the sequence in the film where the hazard emerged and then ended. The six summary eye movement parameters were calculated for both the ‘hazard sequence’ and the ‘non-hazard’ sequence for each film and each participant. The individual differences were then calculated, and these differences were treated as the response variable analysed with a GLM ANOVA. The null hypothesis being no difference in the average value for the eye movement parameters between the patients and controls examined in the two different sequences (F test on the main factor, participant group, from the ANOVA).

A novel ‘point of regard’ analysis was also implemented. The eye tracking sequence is co-registered to the film sequence with each frame of the film equating to nine eye-tracking samples; a mean of these (x,y) points is defined as the ‘point of regard’ for that frame. This is repeated for all the control subjects such that each film frame has a sample of ‘points of regard’ (x,y), one for each control subject, for that particular frame. To quantify the spatial coincidence of the point of regard location for all the control subjects, we calculated the best-fit bivariate contour ellipse (BCE). The BCE area has been previously used to quantify fixation eye movement stability in patients with macular degeneration [22], [26], [27] and to quantify viewing areas for subjects as they watch movies [28]. For our application we implemented the method as described by Goldstein et al [28] but in a novel development we also plotted the ellipse on each frame of the film [29], [30] thus giving a dynamic visual representation of the region of interest for the control subjects. The centre of the BCE represents the mean ‘point of regard’ of the controls, with the spatial extent of the ellipse being one standard deviation from this centre along two principal axes, theoretically affording ‘coverage’ of approximately 67% of the ‘point of regard’ locations in a given frame. The percentage of patients’ points of regard falling inside the BCE should be 67% under a null hypothesis that the glaucomatous patients were, on average, viewing the same parts of the driving scene as the visually healthy subjects.

Monocular visual fields for the patients were merged to form an integrated visual field [19], [31]: a simulated binocular visual field in which patients' best point-by-point monocular sensitivity is used (PROGRESSOR software: Moorfields Eye Hospital, London, UK/Medisoft Ltd., Leeds, UK). The software application developed in this work then aligns and scales a greyscale of this binocular field of view to the gaze point for each patient; this provides a dynamic illustration of a patient's restricted field of view as the film sequence runs, giving an insight into the difficulty of the task from the patient's perspective.

## Results

Data from nine glaucomatous patients and ten age-matched healthy control subjects fulfilled the inclusion criteria used for this study. The mean age of the patients was 67.6 (SD:9.3) years compared to a mean age of 64.4 (SD = 11.4) years for the control subjects (Averages not significantly different; P = 0.52; two sample t-test. Variances not significantly different; P = 0.59; F-test). The self-reported mean number of years actively driving was 29 (SD = 10) and 30 (SD = 12) for the patients and the controls respectively (averages not significantly different; P = 0.86; two sample t-test). The patients had a range of visual field defect severity: average HFA mean defect (MD) was -8.0 (SD = 8.8) dB, -5.4 (SD = 5.7) dB and -8.9 (SD = 8.6) in the right eye, left eye and worse eye, respectively.

A small number of films were missing for the nine patients and ten control subjects because of corrupt data or particularly noisy sequences where more than 60% of the data were removed by the filtering: a threshold used in previous studies.[22] This affected results from one control subject and two glaucomatous patients, but none of these had fewer than 23 films available for analysis. Prior to settling on the numbers for this study, other subjects fulfilling all the other inclusion criteria for the study (2 patients and 2 controls) had several films (more than 12) where a large proportion of data were corrupt, missing, poorly calibrated or had more that 60% removed by the filtering algorithm: these subjects (some of whom had reported difficulty with the task or eye tracking failures) were not included in the final 19 assessed.

**Figure 1:**
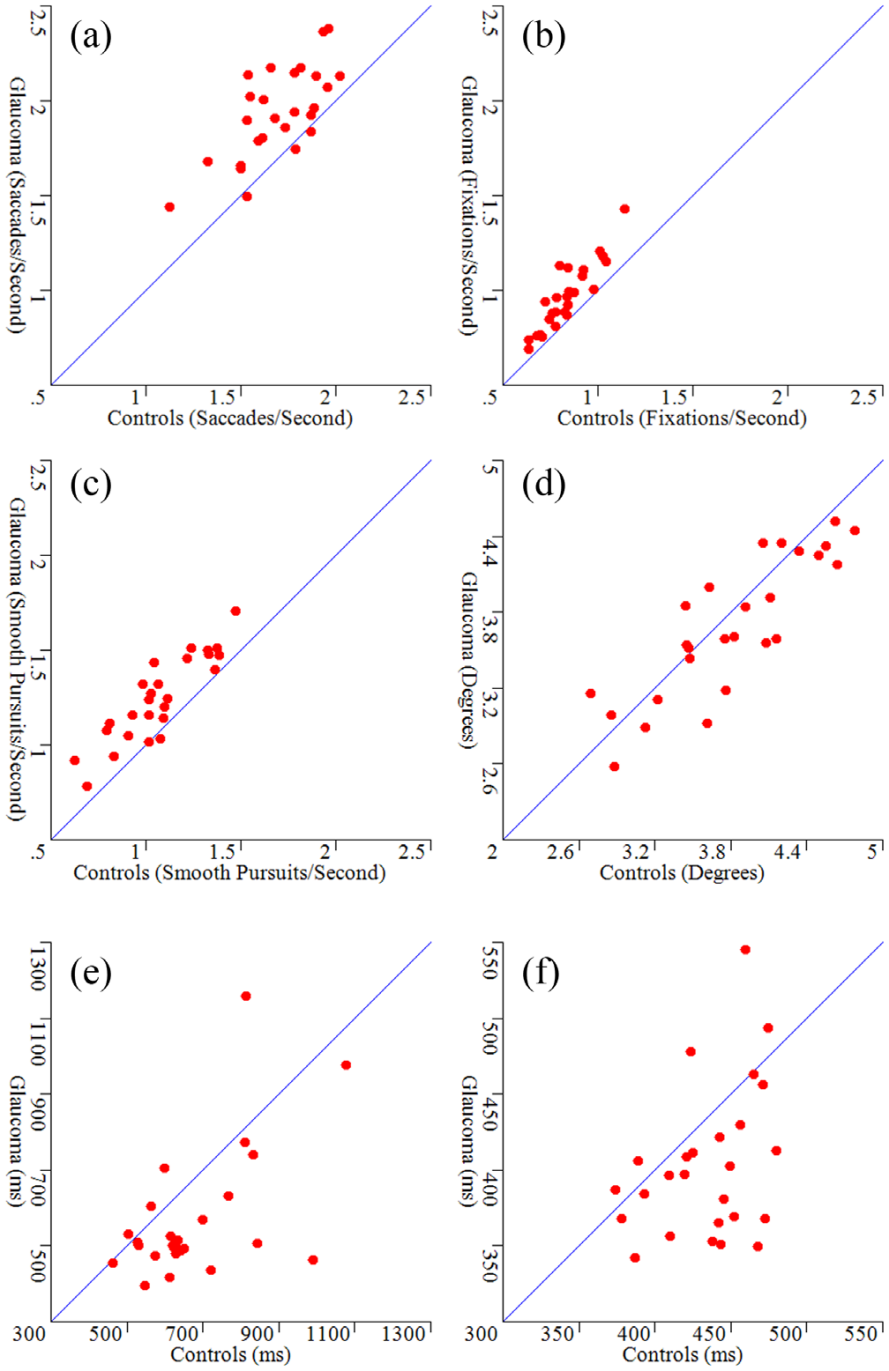
Eye movements for patients and controls. Graphs showing the average response in each eye movement parameter for the patients and the controls with (a) average number of saccades per second; (b) average number fixations per second; (c) number of smooth pursuits per second of film; (d) average saccade amplitude (size in degrees); (e) average fixation duration (ms); and (f) average ‘smooth pursuit’ duration (ms). Each symbol represents the results from one of the 26 films. If there were no differences in the averages then symbols would fall exactly on the line of unity. For example, (a) indicates that the patients made, on average, more saccades across the films than the control subjects, which illustrates the statistically significant effect found in the GLM ANOVA.

Each of the eye movement parameters was assessed in a GLM ANOVA: the departure of the F statistic (on 1 and 25 degrees of freedom) from 1 summarises the extent of a difference in the main factor (patients versus controls) and the P-value refers to the null hypothesis of no effect, or no difference, in that main factor. (No obvious departure from Normality was observed in any of the response variables as assessed by the Kolmogorov-Smirnov test). There was considerable statistical evidence that on average patients made more saccades per second (P<0.001; F_1,25_ = 48.6; mean increase 15.8%; 95% confidence interval [CI]: 9.2 to 22.4%), more fixations per second (P<0.001; F_1,25_ = 53.4; mean increase 16.9%; 95% CI: 11.7 to 22.1%) and more smooth pursuits per second (P<0.001; F_1,25_ = 85.3; mean increase 18.4%; 95% CI: 14.4 to 22.4%) than the control subjects. There was no evidence for a difference in average saccade amplitude (P = 0.12; F_1,25_ = 2.6) There was evidence of average duration of the fixations being shorter in patients (P<0.001; F_1,25_ = 29.6; mean difference 16.5%; 95% CI: 10.0 to 23.0%) compared to controls. There was also evidence of the average duration of smooth pursuits being significantly greater in the controls compared to the patients (P<0.001; F_1,25_ = 22.1; mean difference of 7.4%; 95% CI 3.2 to 11.6%). These results are further illustrated in figure 1: each graph considers a different eye movement parameter, with each point in the graph being the result from each of the 26 films. If the average value for an eye movement parameter for the patients was identical to the controls' then the point would fall exactly on the line of unity. The relative position of the points either below or above this line of unity gives a graphical indication of the magnitude and direction of the experimental effect and illustrates the ANOVA results.

Ten of the films had one hazard, fourteen films had two hazards and two films had three hazards. On average, and across all films, the ‘hazardous’ sections made up 18% (95% CI: 15 to 21%) of the total film sequence. The difference in each of the six eye movement parameters, for each film and for each subject was calculated then used as the response variable in a GLM ANOVA. As before, the F-statistic on the main factor (patients versus controls) was assessed, with the null hypothesis that there was, on average, no effect across the patients and controls between hazard and non-hazard sequences. There was no such significant effect in any of the eye movements across the main factor (F_1,25_ less than 2.92 and P at least >0.10 in all 6 parameters). For example, the average percentage increase in number of saccades per second for the patients compared to controls was 15.7% during the non-hazard sequence (95% CI: 8.9 to 22.5) and 16.3% (95% CI: 6.2 to 26.4 %) during the hazard sequence. This does not support a hypothesis that differences in eye movement measurements between glaucomatous patients and control subjects became more significant or more exaggerated during the ‘hazardous’ parts of the film.

**Figure 2:**
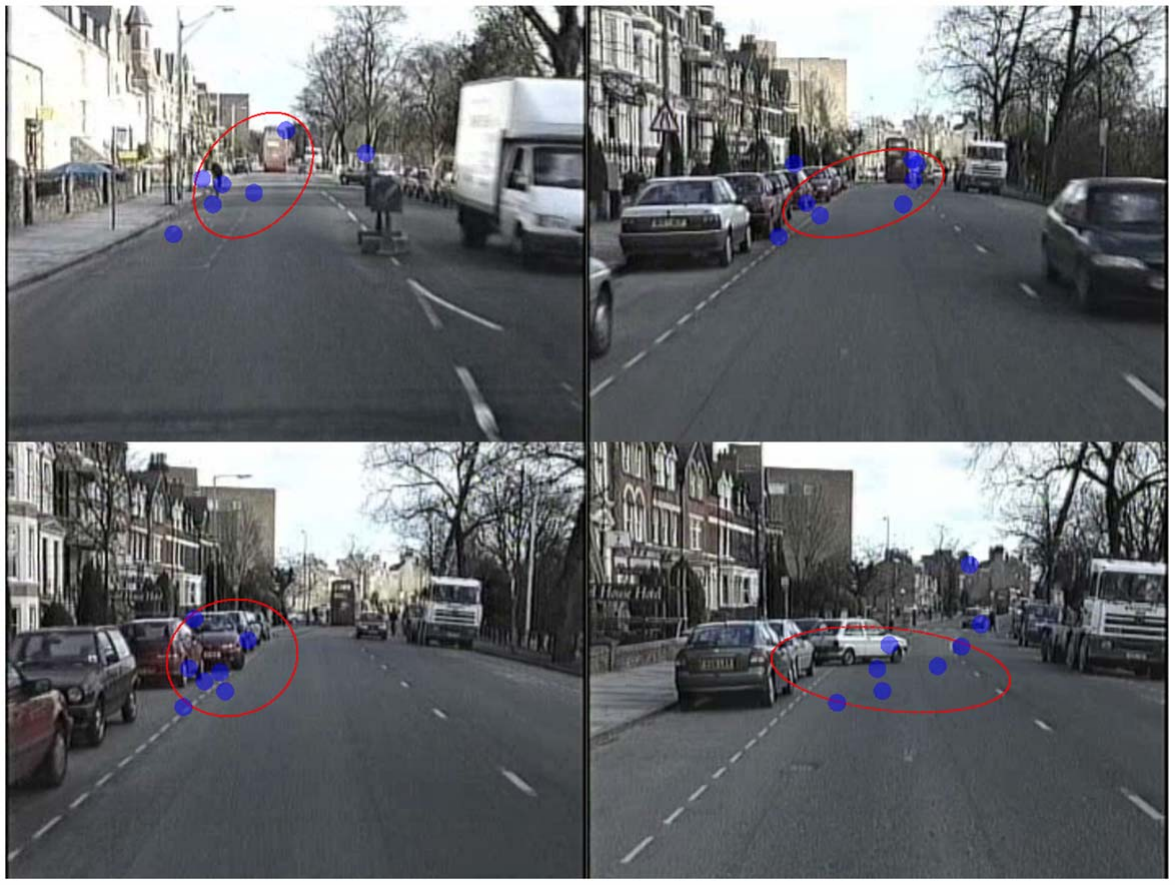
Novel ‘Point of Regard’ Analysis. Examples of the BCE ‘point of regard’ analysis superimposed on frames from some of the HPT films. The centre of the red ellipse represents the mean ‘point of regard’ of the controls with the spatial extent of the ellipse being one standard deviation from this centre along two principal axes, theoretically affording ‘coverage’ of approximately 67% of the ‘point of regard’ locations in a given frame. Note how the location and spatial extent of this ellipse changes with each frame of the film as the control subjects' view is drawn to different aspects of the changing road scene. For each frame the number of patients' points of regard (blue symbols) ‘captured’ by the ellipse can be automatically counted and compared to the expected value under the null hypothesis that the average ‘viewing area’ is the same in the two groups. Note that not all nine patients are recorded in each frame because of blinks, loss of data, and variable responses. Also, note that in the UK vehicles are driven on the left hand side of the road. See Audio/Video - S1.

**Figure 3:**
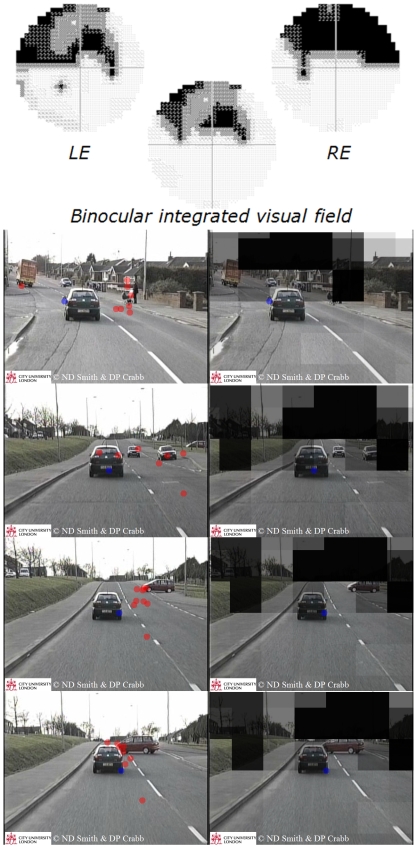
Visual fields, dynamic ‘point of regard’ and HPT films – example. HFA grayscales for monocular (30-2) and binocular integrated visual fields for one of the participants with bilateral glaucoma. The integrated visual field is scaled and superimposed on the point of regard HPT film to give a representation of the restricted field of view. The panels show a montage of selected frames from one of the films. In the top frame, whilst most of visually healthy subjects (red symbols) fixate on the pedestrian with a pram/pushchair, seeing them as a potential hazard, the patient (blue symbol) fails to alter their gaze. Similarly in the next frames most of control subjects quickly move their fixation towards the looming hazard of the car hazardously turning on to the main street from the right intersection. Note that in the UK vehicles are driven on the left side of the road. The patient's view of this hazard is masked by their defect and they do not alter their gaze even at the late stage where the camera car ‘brakes’ to take evasive action (bottom frame). This patient has a binocular visual field that is on the borderline of satisfying the UK guideline for visual field fitness to drive. This montage is shown as a film in Audio/Video - S2 and Audio/Video - S3.

The BCE analysis attempted to compare the overall viewing region of the two groups (figure 2 and Audio/Video - S1). The mean number of points of regard that fell in the BCE was calculated for each patient with the overall average for the glaucomatous group being 62.8% (SD 8.3%). This average was clearly not significantly different from the theoretically expected value (67%) generated from the control group (P = 0.49; one sample t-test). In some films, this average value was as high as 84.9% and in others as low as 56.1% but there did not seem to be any observed pattern of viewing region in the patients departing from that of the controls being associated with the type of driving scene (e.g. rural, urban, divided highway/dual carriageway). In short, participants tended to view the same overall region of the driving scene whether they were glaucomatous or not, but this was on average, and considering the group as a whole. By using the software developed for this study it was possible to follow the viewing pattern of individual patients as they viewed the different films and compare this view with the pattern from all the control subjects simultaneously. Although difficult to quantify, there were some very revealing examples of individual patients clearly not following the control viewing pattern and unambiguous cases where the patient was unaware of emerging hazards. These examples became more informative when the integrated visual field defect of the patient was superimposed on the films giving a perspective of the patient's ‘struggle’ with their restrictive binocular central field defect. One of these cases is shown as a montage in figures 3 and as film clips in Audio/Video - S2, Audio/Video - S3.

## Discussion

These results provide some new evidence that some characteristics of eye movements in glaucomatous patients with binocular visual field defects are different to those of healthy control subjects when performing the HPT. On average, the patients made more saccades and more fixations than controls. With this task we speculate that patients are, unconsciously or otherwise, making more saccades to search the image as a ‘compensation’ for their restricted field of view. Since a binocular visual field defect reduces the amount of visual information available, this might suggest fewer eye movements [32], but this was not the case when patients were viewing these moving images. Our results also indicate that fixation duration was, on average, shorter with patients compared to controls. In contrast, it has been previously shown that fixation duration increases with the size of absolute central scotomas, but this was in a study using artificial scotomas on healthy subjects and a different task (searching a static image) [32]. Others have shown a reduction in fixation duration when an artificial scotoma is used to block foveal vision. [33]

Whilst most of the eye movement measurements were, on average, different between patients and controls, the BCE analysis indicated that patients were, on average, looking at the same areas as control subjects in the HPT movies. Despite the novelty and computational difficulty of this analysis it is probably too insensitive to the subtle departures in patient gaze as compared to the controls. The BCE area was often relatively large, being poorly estimated with so few controls. Moreover, there was no evidence from this sample to support the idea that eye movements become more or less exaggerated in patients compared to controls during the sequence of the film where a hazard was present or emerging. This temporal analysis suffers from the difficulty of the likely weaker estimates of the eye movement measurements during the hazard sequence because they only make up a smaller percentage of the film sequence. Looking at individual cases viewing individual films is helpful but provides little quantitative information about how the patients differ from a ‘normal’ viewer. A better post-hoc analysis might mark out areas in the frames that are of interest, perhaps associated with the looming hazards, and then quantify the patient's gaze falling in these areas. Such an analysis would need larger sample numbers than made available for this study to provide an adequate comparison.

Eye movement studies in ‘real world’ or simulated ‘real world’ environments have tended to focus on experiments with artificial scotomas or, when carried out in patient groups, with retinitis pigmentosa (RP) [34], [35] and age-related macular degeneration (MD) [36]. Few studies have previously examined eye movement behaviour in glaucomatous subjects. An elegant study by Cheong et al [37] examined ‘traffic gap’ judgement and eye movements in subjects with peripheral visual field loss, including three subjects with glaucoma, as compared to healthy subjects. They reported restricted gaze patterns using the BCE approach but little evidence of difference in saccade amplitudes, or fixation duration. Eye tracking experiments by Geruschat et al [38] demonstrated that on average glaucomatous patients (n = 12) had similar fixation patterns as compared to controls when crossing a street. Coeckelbergh et al [39] measured eye movements on a simple static search task and attempted to relate these to an actual on-road driving test in 50 subjects with field loss, some of whom were glaucomatous (number not given in the study). The number of fixations in the search task increased as a function of the size of the peripheral visual field defect, a finding consistent with our results. They reported some association between fixation duration and severity of central field defect but then indicated that eye movement parameters recorded during a simple static search task did not predict viewing behaviour in the actual task of driving. This is not surprising since gaze patterns are intrinsically related to the specific task: their patterns obtained from a static search task are likely to differ from patterns for a ‘driving’ task. Indeed eye movement patterns for the latter have been shown to be extremely complex [40].

The HPT was previously studied as a possible surrogate for the UK visual field standard for fitness to drive [18]. The main measurement considered was the press button response which yielded the percentage of hazards missed and the mean hazard response time. The main finding from this study was that the HPT, when using detection and speed of detection of the hazard, was of limited value when used alone in identifying subjects with visual field loss incompatible with current UK driving standards. Our current interest in the HPT is quite different: for the purpose of our current study, the HPT is used as a realistic, dynamic, visually challenging ‘scene’ whilst eye movements are simultaneously recorded.

As in any case-control study it is prudent to highlight potential confounders. The patients were age-matched to the controls and previous work with the HPT and eye movement analysis in healthy subjects has shown there is little evidence of an age-related difference in HPT performance anyway [41]. It has been previously shown that experienced drivers demonstrate more extensive scanning than the novices in performing the HPT [42], [43]. In this study we only included participants that had driven for at least 5 years, and the patient and control groups were almost identically matched for self reported years of driving experience. Participants were excluded from the original study if they were on any ‘significant medication’ other than that for their glaucoma. Significant medication' included anti-depressants or treatment for diabetes or significant use of β-blocker medication, all of which were deliberately mentioned. However, this was done by self report rather than verification with their general medical practitioner and this might limit the interpretation of the results. Moreover, participants may not have reported use of mild anti-hypertensive or statins or other commonly used drugs in an elderly population. There is, however, no reason to suspect that the glaucomatous group would have had more or less of these participants compared to the age-matched control group.

Other caveats to be considered when interpreting the results from this study include the difficulty in truly identifying and separating the different types of eye movement, especially delineating saccades from smooth pursuits when the participant observes a moving image. This is a challenging task even with modern eye tracking instrumentation. We took the approach of excluding some subjects and removing significant sections of eye movement traces where it was not possible to identify clearly the different movements using an algorithm based on eye movement velocity developed for the purpose. The experiment was not an easy one for some subjects to perform, taking around 30 minutes to view all the film clips, and this probably contributed to the variability in the measurements. Furthermore, the patient group for our study was small, meaning population inference should be treated cautiously, but the sample size was certainly equivalent to those used in many experimental eye movement studies in the literature.

Anecdotally, some patients criticize the current static binocular visual field test (Esterman Test) used to establish legal fitness to drive in the UK for not allowing ‘scanning’ for a target. An eye movement and HPT task might be useful as a test for visual field restriction and fitness to drive. The set up would benefit from films shown on a larger screen, equivalent to that ‘view’ from a vehicle, and an eye tracker device that allows for natural movements. Such a test would not be reliant on press-button defined hazard detection but could simply monitor the patient's natural gaze. A post-hoc analysis of gaze patterns, compared to visually healthy control subjects, as illustrated in this study, could determine if patients ‘saw’ hazards, road/traffic signs and events that might provide evidence for them being visually ‘safe’ drivers. Taken alone it is very unlikely that such an idea would solve the difficulty of determining the vision standard for fitness to drive, the skills for which are so multifactorial that it defies one simple standard. Besides, the low task demand of watching a film may give an impression of competent driving performance that may not be maintained if someone is controlling a vehicle. Nevertheless, given the debate about current visual field standards for driving [44], [45] such an approach may warrant further investigation.

The films showing, frame by frame, a patient's gaze pattern diverging from those of a number of visually healthy subjects provide a revealing insight into the visual impairment caused by glaucoma. This is particularly striking when the individual binocular visual field defects, made dynamic by aligning them to the moving point of regard, are superimposed on the films. One significant output from this work has been the use of these films in educational/awareness programmes for glaucoma in the UK and the subsequent interest they have generated, including a feature on the BBC (British Broadcasting Corporation) News [46].

In conclusion, this exploratory study provides some new evidence that patients with bilateral glaucoma exhibit different eye movement behaviour compared to visually healthy subjects when viewing a driving scene. We hope these results from this exploratory study will at least stimulate investigations into the novel idea that eye movement studies, perhaps coupled with work on patient's adaption to visual field defects, might provide a new ‘window’ into understanding the functional deficits of glaucoma. We speculate that understanding such ‘real world’ visual function deficits in glaucoma is perhaps a first step towards designing appropriate strategies for patient education about the impact of a visual field defect and potential rehabilitation, an overlooked area in this condition as compared, for example, to AMD and RP. Further studies in eye movements and glaucoma are already underway in our laboratory.

## Supporting Information

Audio/Video S1 Novel ‘Point of Regard’ Analysis film. Video example of the BCE ‘point of regard’ analysis superimposed on a HPT film from figure 2. The centre of the red ellipse represents the mean ‘point of regard’ of the controls with the spatial extent of the ellipse being one standard deviation from this centre along two principal axes, theoretically affording ‘coverage’ of approximately 67% of the ‘point of regard’ locations in a given frame.
(9.16 MB AVI)

Audio/Video S2 Participants ‘point of regard’ during HPT film. Video of the HPT film from figure 3 showing the ‘point of regard’ of all the control subjects. The blue point represents the point of regard for a glaucomatous patient Note that in the UK vehicles are driven on the left hand side of the road.
(7.17 MB AVI)

Audio/Video S3 Participants ‘point of regard’ during HPT film showing a patient's ‘view’. Video of the HPT film from figure 3 showing the ‘point of regard’ of the glaucomatous patient in video S2 with their binocular integrated visual field superimposed. The degree of transparency relates to the patients defect in that region, with the least transparent regions being most defective. Note that in the UK vehicles are driven on the left hand side of the road.
(7.48 MB AVI)

## References

[1] Haymes SA, LeBlanc RP, Nicolela MT, Chiasson LA, Chauhan BC (2007). Risk of Falls and Motor Vehicle Collisions in Glaucoma.. Investigative Ophthalmology & Visual Science.

[2] White SC, Atchison KA, Gornbein JA, Nattiv A, Paganini-Hill A (2006). Risk factors for fractures in older men and women: The Leisure World Cohort Study.. Gender Medicine.

[3] Szlyk JPP, Mahler CLMS, Seiple WP, Edward DPMD, Wilensky JTMD (2005). Driving Performance of Glaucoma Patients Correlates With Peripheral Visual Field Loss.. Journal of Glaucoma.

[4] Jampel HD, Friedman DS, Quigley H, Miller R (2002). Correlation of the Binocular Visual Field with Patient Assessment of Vision.. Invest Ophthalmol Vis Sci.

[5] Viswanathan AC, McNaught AI, Poinoosawmy D, Fontana L, Crabb DP (1999). Severity and Stability of Glaucoma: Patient Perception Compared With Objective Measurement.. Arch Ophthalmol.

[6] Friedman DS, Freeman E, Munoz B, Jampel HD, West SK (2007). Glaucoma and Mobility Performance: The Salisbury Eye Evaluation Project.. Ophthalmology.

[7] Nelson PP, Aspinall PP, Papasouliotis OP, Worton BP, O'Brien CMDFF (2003). Quality of Life in Glaucoma and Its Relationship with Visual Function.. Journal of Glaucoma.

[8] Noe G, Ferraro J, Lamoureux E, Rait J, Keeffe JE (2003). Associations between glaucomatous visual field loss and participation in activities of daily living.. Clinical & Experimental Ophthalmology.

[9] Altangerel U, Spaeth GL, Steinmann WC (2006). Assessment of Function Related to Vision (AFREV).. Ophthalmic Epidemiology.

[10] Kotecha A, O'Leary N, Melmoth D, Grant S, Crabb DP (2009). The Functional Consequences of Glaucoma for Eye-Hand Coordination.. Invest Ophthalmol Vis Sci.

[11] Haymes SA, LeBlanc RP, Nicolela MT, Chiasson LA, Chauhan BC (2008). Glaucoma and On-Road Driving Performance.. Invest Ophthalmol Vis Sci.

[12] Johnson CA, Keltner JL (1983). Incidence of visual field loss in 20,000 eyes and its relationship to driving performance.. Arch Ophthalmol.

[13] McGwin G, Mays A, Joiner W, DeCarlo DK, McNeal S (2004). Is Glaucoma Associated with Motor Vehicle Collision Involvement and Driving Avoidance?. Investigative Ophthalmology & Visual Science.

[14] Ang GS, Eke T (2006). Lifetime visual prognosis for patients with primary open-angle glaucoma.. Eye.

[15] Owen VMF, Crabb DP, White ET, Viswanathan AC, Garway-Heath DF (2008). Glaucoma and Fitness to Drive: Using Binocular Visual Fields to Predict a Milestone to Blindness.. Invest Ophthalmol Vis Sci.

[16] Bhargava JS, Patel B, Foss AJE, Avery AJ, King AJ (2006). Views of Glaucoma Patients on Aspects of Their Treatment: An Assessment of Patient Preference by Conjoint Analysis.. Invest Ophthalmol Vis Sci.

[17] Chisholm CM, Rauscher FG, Crabb DP, Davies LN, Dunne MC (2008). Assessing visual fields for driving in patients with paracentral scotomata.. Br J Ophthalmol.

[18] Rauscher F, Chisholm C, Crabb D (2007).

[19] Crabb DP, Fitzke FW, Hitchings RA, Viswanathan AC (2004). A practical approach to measuring the visual field component of fitness to drive.. Br J Ophthalmol.

[20] Grayson G, Sexton B (2002). Hazard Perception Test.. Transport Research Laboratory TRL558.

[21] Smith ND, Chisholm CM, Edgar DF, Barbur JL, Garway-Heath DF (2007). Eye Movement analysis in Glaucoma When Viewing a Driving Hazards Perception Test..

[22] Crossland MD, Rubin GS (2002). The use of an infrared eyetracker to measure fixation stability.. Optometry and Vision Science.

[23] van der Geest J (2002). Recording eye movements with video-oculography and scleral search coils: a direct comparison of two methods.. Journal of Neuroscience Methods.

[24] Hsiao JH, Cottrell G (2008). Two Fixations Suffice in Face Recognition.. Psychological Science.

[25] Stampe DM (1993). Heuristic filtering and reliable calibration methods for video based pupil tracking systems.. Behavior Research Methods, Instruments, and Computers.

[26] Bellmann C, Feely M, Crossland MD, Kabanarou SA, Rubin GS (2004). Fixation stability using central and pericentral fixation targets in patients with age-related macular degeneration.. Ophthalmology.

[27] González E, Teichman J, Lillakas L, Markowitz S, Steinbach M (2006). Fixation stability using radial gratings in patients with age-related macular degeneration.. Can J Ophthalmol.

[28] Goldstein RB, Woods RL, Peli E (2007). Where people look when watching movies: Do all viewers look at the same place?. Computers in Biology and Medicine.

[29] Altman DG (1978). Plotting Probability Ellipses.. Applied Statistics.

[30] Healy HJR (1972). Drawing a Probability Ellipse.. Applied Statistics.

[31] Crabb DP, Viswanathan AC (2005). Integrated visual fields: a new approach to measuring the binocular field of view and visual disability.. Graefe's Archive for Clinical and Experimental Ophthalmology.

[32] Cornelissen FWP, Bruin KJP, Kooijman ACP (2005). The Influence of Artificial Scotomas on Eye Movements during Visual Search.. Optometry & Vision Science.

[33] Henderson JM, McClure KK, Pierce S, Schrock G (1997). Object identification without foveal vision: Evidence from an artificial scotoma paradigm.. Perception & Psychophysics.

[34] Vargas-Martin F, Peli E (2006). Eye Movements of Patients with Tunnel Vision While Walking.. Invest Ophthalmol Vis Sci.

[35] Turano KA, Geruschat DR, Baker FH, Stahl JW, Shapiro MD (2001). Direction of Gaze while Walking a Simple Route: Persons with Normal Vision and Persons with Retinitis Pigmentosa.. Optometry and Vision Science.

[36] Crossland MD, Rubin GS (2006). Eye movements and reading in macular disease: further support for the shrinking perceptual span hypothesis.. Vision Res.

[37] Cheong AMY, Geruschat DR, Congdon N (2008). Traffic Gap Judgment in People with Significant Peripheral Field Loss.. Optometry and Vision Science.

[38] Geruschat DR, Hassan SE, Turano KA, Quigley HA, Congdon NG (2006). Gaze Behavior of the Visually Impaired During Street Crossing.. Optometry and Vision Science.

[39] Coeckelbergh TRM, W.Cornelissen F, H.Brouwer W, C.Kooijman A (2002). The effect of visual field defects on eye movements and practical fitness to drive.. Vision Research.

[40] Land MF, Lee DN (1994). Where we look when we steer.. Nature.

[41] Underwood G, Phelps N, Wright C, Loon E, Galpin A (2005). Eye fixation scanpaths of younger and older drivers in a hazard perception task.. Ophthalmic and Physiological Optics.

[42] Underwood G, Chapman P, Bowden K, Crundall D (2002). Visual search while driving: skill and awareness during inspection of the scene.. Transportation Research Part F: Traffic Psychology and Behaviour.

[43] Underwood G, Chapman P, Brocklehurst N, Underwood J, Crundall D (2003). Visual attention while driving: sequences of eye fixations made by experienced and novice drivers.. Ergonomics.

[44] Kotecha A, Spratt A, Viswanathan A (2008). Visual function and fitness to drive.. Br Med Bull.

[45] Westlake W (2000). Another look at visual standards and driving.. BMJ.

[46] BBC (2008). http://news.bbc.co.uk/1/hi/health/7660570.stm.

